# Genomic Tools in Pea Breeding Programs: Status and Perspectives

**DOI:** 10.3389/fpls.2015.01037

**Published:** 2015-11-27

**Authors:** Nadim Tayeh, Grégoire Aubert, Marie-Laure Pilet-Nayel, Isabelle Lejeune-Hénaut, Thomas D. Warkentin, Judith Burstin

**Affiliations:** ^1^INRA, UMR1347 AgroécologieDijon, France; ^2^INRA, UMR1349 Institut de Génétique Environment et Protection des PlantesLe Rheu, France; ^3^INRA, USC Institut Charles Viollette-Adaptation au Froid du PoisEstrées-Mons, France; ^4^Crop Development Centre, College of Agriculture and Bioresources, University of SaskatchewanSaskatoon, SK, Canada

**Keywords:** pea (*Pisum sativum* L.), breeding targets, genetic diversity, genomic resources, genotyping platforms, genetic maps, QTL and association mapping

## Abstract

Pea (*Pisum sativum* L.) is an annual cool-season legume and one of the oldest domesticated crops. Dry pea seeds contain 22–25% protein, complex starch and fiber constituents, and a rich array of vitamins, minerals, and phytochemicals which make them a valuable source for human consumption and livestock feed. Dry pea ranks third to common bean and chickpea as the most widely grown pulse in the world with more than 11 million tons produced in 2013. Pea breeding has achieved great success since the time of Mendel's experiments in the mid-1800s. However, several traits still require significant improvement for better yield stability in a larger growing area. Key breeding objectives in pea include improving biotic and abiotic stress resistance and enhancing yield components and seed quality. Taking advantage of the diversity present in the pea genepool, many mapping populations have been constructed in the last decades and efforts have been deployed to identify loci involved in the control of target traits and further introgress them into elite breeding materials. Pea now benefits from next-generation sequencing and high-throughput genotyping technologies that are paving the way for genome-wide association studies and genomic selection approaches. This review covers the significant development and deployment of genomic tools for pea breeding in recent years. Future prospects are discussed especially in light of current progress toward deciphering the pea genome.

## Introduction

Thanks to significant technological breakthroughs, pea genetics is rapidly evolving from conventional to large-scale molecular-assisted approaches to uncover the molecular bases of important traits and enhance breeding. Several reviews have been published recently that dealt with pea systematics, seed quality, and breeding (Burstin et al., 2011; Dahl et al., 2012; Bohra et al., 2014; Arnoldi et al., 2015; Smýkal et al., 2015; Varshney et al., 2015; Warkentin et al., 2015). The present review focuses on the genomic toolkit that was developed recently in pea thanks in part to next-generation sequencing technologies. This includes transcriptome, genotyping, and mapping resources that will pave the way to renewed pea breeding programs.

## Economic importance, nutritive value, growing regions

Pea (*Pisum sativum* L.) is a major cool-season pulse crop and an essential component of sustainable cropping systems (Nemecek et al., 2008; Duc et al., 2010; Jensen et al., 2012). Significant agro-ecological services linked with its ability to develop symbiotic nitrogen fixation as well as its role as a break crop for pest and pathogen pressure reduction have been described (Nemecek and Kägi, 2007; Hayer et al., 2010; Macwilliam et al., 2014). In 2013, the vegetable pea production amounted to 17.43 Mt worldwide (FAOSTAT)^1^ and dry pea represented the third most important pulse crop production after common bean and chickpea with 11.16 Mt produced worldwide (FAOSTAT).

Pea seeds are an important source of proteins and provide an exceptionally varied nutrient profile (for a review, Burstin et al., 2011): major constituents are starch (from 18.6 to 54.1%) and proteins (15.8–32.1%), followed by fibers (5.9–12.7%), sucrose (1.3–2.1%), and oil (0.6–5.5%). Seeds also contain minerals, vitamins, and micro-nutrients such as polyphenolics, saponins, α-galactosides, and phytic acids whose health-promoting effects are being tested (Bastianelli et al., 1998; Mitchell et al., 2009; Dahl et al., 2012; Marles et al., 2013; Arnoldi et al., 2015). Peas enter in human nutrition in a wide diversity of forms: fresh seedlings, immature pods, and seeds provide a green vegetable, and whole or ground dry seeds are cooked in various dishes. High quality starch, protein, or oligoside isolates are being extracted from dry pea seeds and whole seed structural and functional characteristics have been assessed for food improvement (Brummer et al., 2015). Because dry seeds contain little anti-nutritional factors, they are also introduced as a protein source mainly in monogastric diets without affecting growth and production traits (Stein et al., 2006; Laudadio et al., 2012; Dotas et al., 2014). Pea hay is used as fodder in ruminant diets (Bastida Garcia et al., 2011).

Pea is mainly cultivated in temperate regions of the world on well-drained and fertile soils. However, being distributed over all continents, the pea production area is characterized by a large range of pedo-climatic conditions. Indeed, China is the largest producer of vegetable peas (10.60 Mt, FAOSTAT) followed by India (4 Mt). Canada is the main producer of dry peas (3.85 Mt) followed by China (1.6 Mt), the Russian Federation (1.35 Mt), USA (0.71 Mt), India (0.60 Mt), France (0.50 Mt), and Ethiopia (0.40 Mt).

## Variability of germplasm used for breeding

Pea was domesticated by Neolithic farmers in the Fertile Crescent some 10,000 years ago (Willcox et al., 2009; Weiss and Zohary, 2011; Smýkal, et al., 2014). Pea then spread rapidly toward south-west Asia, the Mediterranean basin, and Europe (Zohary, 1999). Probably linked with their large range of cultivation and the diversity of their use as food, feed, or fodder, pea landraces and varieties now exhibit an incredible diversity of forms and growing types, adapted to diverse environments, cropping systems, and end-uses (Burstin et al., 2015). This vast diversity of cultivated forms is the major reservoir for present crop improvement. Different types of pea varieties have been developed for vegetable pea production, varying at major genes controlling seed and plant traits. For example, wrinkled seeds are associated with significant changes of seed composition, linked with starch synthesis modification (Wang et al., 2003). Various types of dry peas are also available that differ by their cotyledon color, plant architecture, or flowering time. In addition to this cultivated reservoir of diversity, wild peas can be crossed with cultivated peas. Ben-Ze'Ev and Zohary (1973) showed that chromosomal rearrangements among accessions from the different *Pisum* species and subspecies could cause partial sterility in hybrids. Recently, Bogdanova et al. (2014) have identified a nucleo-cytoplasmic incompatibility between a *P. sativum elatius* accession and cultivated peas. However, within the *Pisum* genus, wild *P. fulvum*, wild subspecies *P. sativum elatius*, and *P. sativum humile* as well as *P. abyssinicum*, a taxon cultivated in Ethiopia, are in most cases inter-crossable with *P. sativum sativum* as long as the cultivated pea is used as female donor (Ben-Ze'Ev and Zohary, 1973; Ochatt et al., 2004). Different authors have thus used *P. fulvum* as well as wild *P. sativum* subspecies as a source of alleles for important breeding traits, such as resistance to various fungal diseases (Barilli et al., 2010; Fondevilla et al., 2011; Jha et al., 2012) or to *Bruchus pisorum* L. (Clement et al., 2009).

## Target traits and achievements through conventional breeding

Continued grain yield improvement is necessary for pea to remain an attractive option compared to cereals and oilseeds in crop rotations. Improving yield involves addressing many biotic and abiotic stresses, using a large set of strategies including diverse germplasm as parents, making many crosses, selecting for major gene traits under conditions conducive to selection, and yield testing of a large number of breeding lines. These stresses are specific to each growing region and/or growing type. However, fungal diseases are the major biotic stress in most cases, followed by various insects, viruses, and parasitic plants such as broomrape. Drought and heat stress at flowering are the main abiotic stresses, while frost, salinity, and early season flooding are diversely important according to growing types.

Important achievements were obtained in pea cultivars through conventional breeding over the past 20 years. Yield gains of approximately 2% per year have been achieved (Warkentin et al., 2015). Lodging resistance has been improved through deployment of the *afila* gene for semi-leafless type (Kujala, 1953; Goldenberg, 1965) and secondarily through selection for increased stem strength (Banniza et al., 2005). Powdery mildew resistance based on the single recessive gene *er-1* (Harland, 1948) has been widely deployed. Partial resistance to the Ascochyta blight complex has been achieved through pyramiding of genes with minor effects (Kraft et al., 1998). Resistance to pea weevil (*Bruchus pisorum* L.) identified in the secondary gene pool (*P. fulvum*) (Clement et al., 2002) was transferred into cultivated pea through backcrossing (Clement et al., 2009; Aryamanesh et al., 2012). Cultivars adapted to winter sowing have been developed and deployed in Europe and north-west USA giving the potential for better yields because of a longer growing season, higher biomass production, and earlier maturity to avoid late season drought and heat stress (Hanocq et al., 2009). The introgression of the *Hr* allele which delays flower initiation until after the main winter freezing periods have passed (Lejeune-Hénaut et al., 2008) permitted to obtain some cultivars with notably improved winter hardiness. Field pea production for whole seed food markets requires appropriate seed visual quality. Quantitative inheritance, transgressive segregation, and moderately high heritability were observed for seed color, shape, and surface dimpling (Ubayasena et al., 2011) allowing for good progress in breeding. Seed protein concentration has been maintained in pea cultivars, even though overall seed yield has increased (Jha et al., 2013).

In some cases, achievements are at an earlier stage of deployment. Useful germplasms such as sources of resistance to various biotic and abiotic stresses have been identified and are currently being evaluated and introgressed. Research in France and USA has led to the identification and introgression of useful variation for resistance to *Aphanomyces* root rot; partial resistance controlled by several quantitative trait loci (QTLs) is being deployed (Pilet-Nayel et al., 2002, 2005; McGee et al., 2012). Improved stress tolerance has been identified in landrace accessions for toxicity to boron (Bagheri et al., 1994), salinity (Leonforte et al., 2013a), iron deficiency (Kabir et al., 2012), and for heat tolerance during flowering (Petkova et al., 2009). Selection for major gene resistance to pea seed-borne mosaic virus and potyviruses is now incorporated into breeding strategies (van Leur et al., 2007). Diversity in pea seed micronutrient concentration (Ray et al., 2014) and an approach to improving iron bioavailability for humans (Liu et al., 2015) have been described.

Future targets in pea breeding include (i) the optimization of pea interactions with Rhizobia, Mycorrhiza and other beneficial microorganisms in view of crop resilience against stresses, (ii) the adaptation of plant morphology and phenology to novel cropping systems, and (iii) the adaptation of seed composition to novel end-use application possibilities.

## Available genomic resources in pea

The pea genome is organized in 7 pairs of chromosomes (2*n* = 2*x* = 14). Its haploid size is estimated at 4.45 Gb (Dolezel et al., 1998; Dolezel and Greilhuber, 2010; Praca-Fontes et al., 2014). It is largely dominated by mobile elements, mainly of the Ty3/gypsy family (Macas et al., 2007). This large genome size and high transposable element content have undoubtedly contributed to delay the development and availability of genomic tools in pea. Recently, several national and international programs have developed diverse valuable genomic resources by taking advantage of cutting-edge sequencing and genotyping technologies. These programs indicate the determination of the pea community to make rapid progress toward targeted and efficient molecular breeding exploiting the rich diversity of pea germplasm and its wild relatives. An international consortium has been initiated in order to generate the full-sequence of the pea genome (Madoui et al., 2015).

Developing wide collections of mapped and easy-to-use molecular markers is among the first steps of gene tagging and gene introgression strategies. Pea genetic maps started to be developed early: Wellensiek (1925) constructed a six-linkage group (LG) map and Lamprecht (1948) published a full map with 7 LGs (see Rozov et al., 1999, for review). Later on, various marker types were developed and numerous linkage maps originating from intra- or inter-subspecific crosses have been generated (Table 1). Decisive progresses were achieved with the availability of mapped SSR and SNP markers. Thanks to their multi-allelic nature, genomic (Ford et al., 2002; Loridon et al., 2005; Sun et al., 2014) and EST-based SSR markers (Burstin et al., 2001; Gong et al., 2010; De Caire et al., 2012; Kaur et al., 2012; Mishra et al., 2012) have been widely used for studying germplasm diversity (Baranger et al., 2004; Smýkal et al., 2008; Zong et al., 2009; Sarikamis et al., 2010) and bridging between different genetic maps. Today, SNPs are the markers of choice because of their abundance, easy-scoring, and amenability to high-throughput genotyping. SNPs were identified based on sequencing data from 4 (Leonforte et al., 2013b), 6 (Sindhu et al., 2014), 8 (Duarte et al., 2014), and up to 16 (Tayeh et al., 2015a) pea genotypes. Illumina GoldenGate (Deulvot et al., 2010; Leonforte et al., 2013b; Duarte et al., 2014; Sindhu et al., 2014), Infinium (Tayeh et al., 2015a), and Sequenom MassARRAY (Cheng et al., 2015) platforms have been deployed for SNP genotyping (Table 2). In total, at least 52 genetic maps have been constructed for different F_2_ or recombinant inbred line (RIL) populations, comprising up to 8503 markers (Table 1). Consensus maps have been built in order to offer higher mapping resolution and better genome coverage (Table 1). These maps combine molecular data from 2 (Aubert et al., 2006; Hamon et al., 2011), 3 (Loridon et al., 2005; Sudheesh et al., 2014), 4 (Hamon et al., 2013; Duarte et al., 2014), 5 (Sindhu et al., 2014), 6 (Bordat et al., 2011), or 12 (Tayeh et al., 2015a) populations.

**Table 1:** **Available individual and consensus genetic maps constructed for bi-parental populations and quantitative trait loci positioned on these maps**.

**Original cross**	**# ind**.	**# LGs**	**# markers**	**Map length (cM)^§^**	**Mapped traits**	**References**
**(A) F2 POPULATIONS**						
Erygel × 661	174	12	69	550	Plant height, flowering time, number of nodes, resistance to Ascochyta blight	Dirlewanger et al., 1994
JI1089 × JI296	–	–	–	–	Leaf resistance to *Mycosphaerella pinodes*	Clulow et al., 1991
Primo × OSU442-15	227	11	108	1369^§^	Dry seed weight, seed color, seed yield, yield components, flowering nodes, total node number	Timmerman-Vaughan et al., 1996, 2005; McCallum et al., 1997
Vinco × Hurst's Greenshaft	–	–	–	–	Resistance to *Pseudomonas syringae* pv. *pisi*	Hunter et al., 2001
Partridge × Early Onward	–	–	–	–	Resistance to *Pseudomonas syringae* pv. *pisi*	Hunter et al., 2001
G0003973 × G0005527	190	11	157	1518	–	Sun et al., 2014
A26 × Rovar	148	13	99	930^§^	Ascochyta blight resistance	Timmerman-Vaughan et al., 2004
NGB5839 × JI1794^a^	92	–	–	–	Node of flower initiation	Weller et al., 2012
IFPI3260 × IFPI3251	94	9	146	1283.3	Resistance to *Uromyces pisi* (Pers.) Wint.	Barilli et al., 2010
**(B) RIL POPULATIONS**						
Puget × 90–2079	127	13	377	1523	Partial Resistance to *Aphanomyces euteiches*	Pilet-Nayel et al., 2002, 2005; Loridon et al., 2005; Hamon et al., 2013
A88 × Rovar	133	11	96	1050^§^	Resistance to Ascochyta blight field epidemics, plant reproductive maturity	Timmerman-Vaughan et al., 2002
Carneval × MP1401	88	10	207	1274^§^	Lodging reaction, Plant height, Resistance to Mycosphaerella blight, grain yield, seed protein concentration, days to maturity	Tar'an et al., 2003; Tar'An et al., 2004
Wt11238 × Wt3557	110	11	91	853	Yield components, seed protein content	Krajewski et al., 2012
Wt10245 × Wt11238	101	12	191	1086	Stem length, internode number, yield components, seed protein content	Irzykowska et al., 2002; Irzykowska and Wolko, 2004; Krajewski et al., 2012
JI296 × DP	135	7	5277	552.2^§^	Resistance to *Mycosphaerella pinodes*, plant height, flowering date	Prioul et al., 2004; Loridon et al., 2005; Tayeh et al., 2015a
Champagne × Terese	164	7	7072	888.2^§^	Photoperiod response, frost tolerance	Loridon et al., 2005; Aubert et al., 2006; Lejeune-Hénaut et al., 2008; Dumont et al., 2009; Tayeh et al., 2015a
VavD265 × Cameor	211	7	6952	752.6^§^	Seed protein quality/quantity (PQL)	Bourgeois et al., 2011; Tayeh et al., 2015a
Ballet × Cameor	207	7	6796	854.5^§^	Flowering time, leaf appearance rate, radiation use efficiency, leaf area, leaf chlorophyll content, shoot length, seed weight, plant N nutrition indices (biomass, nitrogen content, symbiotic N fixation and nodule efficiency), seed protein quality/quantity	Bourion et al., 2010; Bourgeois et al., 2011; Tayeh et al., 2015a
VavD265 × Ballet	211	7	6188	850.1^§^	Seed protein quality/quantity	Bourgeois et al., 2011; Tayeh et al., 2015a
Cameor × Melrose	120	7	8503	736.3^§^	–	Tayeh et al., 2015a
Kazar × Cameor	84	7	7013	700.7^§^	–	Tayeh et al., 2015a
Kazar × Melrose	118	7	3917	682.5^§^	–	Tayeh et al., 2015a
China × Cameor	129	7	7737	833.1^§^	Flowering time, winter frost damage, branching type, leaf area, leaf chlorophyll content, plant height, yield components, plant biomass, harvest index and seed protein content	Deulvot et al., 2010; Klein et al., 2014; Tayeh et al., 2015a
Cameor × Sommette	144	7	5537	769.1^§^	–	Tayeh et al., 2015a
Cameor × Cerise	120	7	7206	523.8^§^	–	Tayeh et al., 2015a
Baccara × PI180693	178	7	4620	705.2^§^	Aphanomyces root rot resistance, earliness at flowering	Hamon et al., 2011, 2013; Duarte et al., 2014; Tayeh et al., 2015a;
Térèse × K586^b^	139	7	249	1113	Yield components, seed protein content, number, and volume of cotyledon cells, flowering time, plant height, number of basal branches, plant biomass and nitrogen content, nitrogen nutrition index, harvest index	Laucou et al., 1998; Loridon et al., 2005; Aubert et al., 2006; Burstin et al., 2007
Shawnee × Bohatyr	187	8	272	1716	Seed Mineral Content, partial resistance to Fusarium wilt Race 2	Loridon et al., 2005; McPhee et al., 2012
Orb × CDC Striker	90	7	431	–	Cotyledon bleaching resistance, visual seed quality traits	Ubayasena et al., 2010, 2011; Sindhu et al., 2014
Alfetta × CDC Bronco	120	11	223	450	Visual seed quality traits	Ubayasena et al., 2011
Kaspa × Parafield	134	9	868	1916	Salinity tolerance at the seedling stage	Leonforte et al., 2013b
DSP × 90–2131^c^	111	9	168	1046	Earliness at flowering, plant height, Aphanomyces root rot resistance	Hamon et al., 2013
Kaspa × Yarrum	106	13	821	1910	Powdery mildew resistance	Sudheesh et al., 2014
Kaspa × ps1771	106	9	852	1545	Powdery mildew resistance, boron tolerance	Sudheesh et al., 2014
JI15 × JI399	77	9	348	1400^§^	Resistance genes (*Ppi1* and *Ppi2*) to *Pseudomonas syringae* pv *pisi*	Ellis et al., 1992; Hall et al., 1997; Hunter et al., 2001
JI281 × JI399	53	10	421	2300^§^	Resistance genes (*Ppi2*) to *Pseudomonas syringae* pv *pisi*	Ellis et al., 1992; Hall et al., 1997; Hunter et al., 2001
JI15 × JI1194	–	10	–	–	–	Ellis et al., 1992; Hall et al., 1997
JI15 × JI61	–	13	–	–	–	Ellis et al., 1992; Hall et al., 1997
JI1201 × JI813	–	3	–	–	–	Ellis et al., 1992; Hall et al., 1997
Carrera × CDC Striker	134	–	245	–	–	Sindhu et al., 2014
1-2347-144 × CDC Meadow^d^	163	7	367	437.2	phytic acid-phosphorus concentration	Sindhu et al., 2014; Shunmugam et al., 2015
Alfetta × P651	144	–	303	–	–	Sindhu et al., 2014
“Afghanistan” (*sym2*) × A1078-239	19	–	–	–	Tolerance to Fusarium root rot	Weeden and Porter, 2007
CMG × PI 220174	225	–	–	–	Tolerance to Fusarium root rot	Weeden and Porter, 2007
Carman × Reward^e^	71	–	–	–	Resistance to Fusarium root rot	Feng et al., 2011
HUVP 1 × FC 1	136	7	57	634	Resistance to *Uromyces fabae* (Pers.) de-Bary	Rai et al., 2011
MN313 × OSU1026	45	–	–	–	Tolerance to *Aphanomyces euteiches*	Weeden et al., 2000
Baccara × 552^f^	178	–	–	–	Aphanomyces root rot resistance, earliness at flowering	Hamon et al., 2011, 2013
P665 × Messire	362	8	414	1188	Resistance to *Mycosphaerella pinodes, Orobanche crenata, Pseudomonas syringae*, earliness, root length, aerial biomass, drought tolerance	Valderrama et al., 2004; Fondevilla et al., 2008, 2011, 2012; Carrillo et al., 2012; Iglesias-García et al., 2015
MN313 × JI 1794	47	9	242	–	Pod dehiscence	Weeden et al., 2002
JI 1794 × Slow	53	–	–	1289	Tolerance to *Fusarium solani* f. sp. Pisi, Pod dehiscence, dry seed weight	Timmerman-Vaughan et al., 1996; Weeden et al., 1998, 2002; Hance et al., 2004
Pennant × ATC113	270	9	155	2686	*Bruchus pisorum* resistance	Byrne et al., 2008; Aryamanesh et al., 2014
**(C) CONSENSUS MAPS BASED ON RIL POPULATIONS**						
Terese × K586; Champagne × Terese; Shawnee × Bohatyr	490	7	462	1430^§^	–	Loridon et al., 2005
Terese × K586; Champagne × Terese	303	7	363	1458^§^	–	Aubert et al., 2006
Terese × K586; Terese × Champagne; China × Cameor; Cameor × VavD265; Cameor × Ballet; Ballet × VavD265	1022	7	536	1389	–	Bordat et al., 2011
Baccara × PI 180693; Baccara × 552	356	7	224	1652	–	Hamon et al., 2011
Baccara × PI 180693; Baccara × 552; DSP × 90-2131; Puget × 90-2079	594	7	619	1513	–	Hamon et al., 2013
Orb × CDC Striker; Carrera × CDC Striker; 1-2347-144 × CDC Meadow; Alfetta × P651; China × Cameor	586	7	939	771.6	–	Sindhu et al., 2014
JI296 × DP; Champagne × Terese; China × Cameor; Baccara × PI180693	360	7	2070	1255	–	Duarte et al., 2014
Kaspa × Yarrum; Kaspa × ps1771; Kaspa × Parafield	–	7	764	2555	–	Sudheesh et al., 2014^g^
Champagne × Terese; VavD265 × Cameor; Ballet × Cameor; VavD265 × Ballet; Cameor × Melrose; Kazar × Cameor; Kazar × Melrose; China × Cameor; Cameor × Sommette; Cameor × Cerise; Baccara × PI180693; JI296 × DP	1384	7	15079	794.9^§^	–	Tayeh et al., 2015a

a NGB5839 is an isogenic dwarf derivative of cv. Torsdag that carries a mutation in the gibberellin biosynthesis gene LE; line JI1794 is a representative accession of the northern race of Pisum sativum var. humile.

b K586 is a ramified mutant obtained from “Torsdag” (Laucou et al., 1998).

c DSP, Dark Skin Perfection.

d Line 1-2347-144 was obtained from CDC Bronco through mutagenesis; it retains the majority of the traits of this latter (Warkentin et al., 2012).

e Reciprocal crosses between Carman and Reward were made and seeds from both reciprocal crosses were used to develop an F_8_ RIL.

f 552 is a garden pea breeding line derived from the 8th cycle of a recurrent selection program conducted for Aphanomyces root rot resistance (Hamon et al., 2011).

g A total of 535 SNP markers from this map originates from transcript sequences common with the SNP markers from Duarte et al. (2014): merging the two datasets resulted in a 7-LG 2028-marker 2387-cM consensus map.

§ cM Haldane.

AFLP, amplified fragment length polymorphism; RFLP, random fragment length polymorphism; RAPD, random amplified polymorphic DNA; SSR, simple sequence repeat; STS, sequence tagged site; ISSR, inter simple sequence repeat; CAPS, cleaved amplified polymorphic site; HRM, high-resolution melting; SNP, single nucleotide polymorphism.

**Table 2:** **Recently-developed resources in pea: transcriptome and whole-genome sequencing data, SNP collections, and genotyping platforms**.

	**References**	**Plant material**			**Sequencing**	**Read assembly**					**Genotyping**						
		**Genotype(s)^a^**	**Organ(s)/ Tissue(s)**	**Contrasted growth condition(s) (if any)**	**Sequencing Platform**	**# reads for assembly**	**Assembler**	**# contigs and singletons**	**Mean contig length (bp)**	**Data accessibility**	**References**	**Platform**	**Marker types**	**Reference genotype for SNP calling**	**# robust filtered SNPs**	**# markers on the genotyping array**	**# high-quality markers**
Gene-specific	Aubert et al., 2006	Cameor, Ballet, Champagne, Kazar, Melrose, VavD265, K586, Térèse, other sources	Leaf	–	Sanger	–	–	–	–	–	Deulvot et al., 2010	GoldenGate	Genomic, EST or cDNA-derived SNP	–	520	384	325
											Cheng et al., 2015	iPLEX Gold platform	SNP from Deulvot et al., 2010	–	–	384	340
Transcriptome	Franssen et al., 2011	Little Marvel	Cotyledon, seedling, hypocotyl, epicotyl, leaf, flower^N^	With or without 6-h light exposure of the seedlings	454 Roche GS FLX or GS20	2,209,735	MIRA	128,767 contigs and 195,661 singletons^b^	324	Transcriptome Shotgun Assembly Archive of GenBank; accession numbers: JI896856 - JI981123	–	–	–	–	–	–	–
	Kaur et al., 2012	Parafield, Yarrum, Kaspa, 96-286	Seed, root, stem, leaf, flower, pod	–	454 Roche GS FLX Titanium	687,200	Next Gene software	13,583 contigs and 57,099 singletons^c^	719	Transcriptome Shotgun Assembly Archive of GenBank; accession numbers: JR950756-JR964200	Leonforte et al., 2013b	GoldenGate	Transcript-based SNP	Consensus reference	956	768	705
	Duarte et al., 2014	Lumina, Hardy, Panache, Rocket, Kayanne, Terese, Cherokee, Champagne	Seedlings^N^	–	454 Roche GS FLX Titanium	3,042,418 (267,463–458,682 total reads per cultivar)	MIRA (“est” mode)	68,850 contigs^d^	842	Transcriptome Shotgun Assembly of GenBank under the accession GAMJ00000000	Duarte et al., 2014	GoldenGate	Transcript-based SNP	Consensus reference	35,455	1920	1620
	Sindhu et al., 2014^e^	CDC Bronco, Alfetta, Cooper, CDC striker, Nitouch, Orb, P651, and PI358610^f^	Seed, seedling, leaf, stem, flower	Normal or dark conditions for seedlings grown in Petri dishes	454 Roche GS FLX Titanium	4,008,648 (520,797–593,701 total 454 reads per cultivar)	NGen (DNAStar) software	29,725 contigs^g^	–	NCBI-NIH Short Read Archive, BioProject ID: PRJNA237996	Sindhu et al., 2014	GoldenGate	Transcript-based SNP	CDC Bronco	8822	1536	1491
	Ferraro et al., 2014	Courier	Seed coat	–	454 Roche GS FLX Titanium	40,903	MIRA	5766 contigs and 10,506 singletons	–	–	–	–	–	–	–	–	–
	Alves-Carvalho et al., 2015	Cameor	Seed, root, nodule, stem, apical node, leaf, peduncule, tendril, flower	Hydroponic or aeroponic growth conditions with either high or low-nitrogen supply	Illumina Genome Analyzer II or HiSeq2000	1,018,751,326	Velvet-Oases and TGICL++	46,099 contigs^h^	1199	http://bios.dijon.inra.fr/FATAL/cgi/pscam.cgi.	–	–	–	–	–	–	–
Whole-genome	Burstin et al., 2014; Tayeh et al., 2015a	Cameor, Ballet, Cerise, Champagne, China, JI281, Kazar, Melrose, PI180693, Serpette d'Auvergne, Sommette, TIL336/11, VavD265, JI1703, JI2202, DCG076^i^	Leaf	–	Illumina HiSeq2000	184,764,428–1,061,737,954 total reads per accession^j^	hku-IDBA	181,125 contigs	756	http://www.ncbi.nlm.nih.gov/bioproject/PRJNA285605/	Tayeh et al., 2015a	Infinium	Gene-space assembly-derived SNP	Cameor	248,617	15,000	13,156

a In all cases, separate sequencing of cDNA libraries from different genotypes were undertaken.

b A second pass assembly is described with 81,449 total unigenes but it lacks about a third of contig annotations obtained for the first pass assembly.

c A total of 45,161 overlapping hits with contigs/singletons from Franssen et al. (2011) were identified (10,832 contigs [24%] and 34,329 singletons [76%]).

d 70,337 contigs (86%) out of 81,449 from Franssen et al. (2011) and 12,776 (95%) out of 13,445 from Kaur et al. (2012) were reported to have a hit against assemblies from Duarte et al. (2014).

e A 3′ transcript profiling was performed: 3′-anchord cDNA libraries were generated and sequenced.

f P651 and PI358610 are wild accessions: P651 (P. fulvum) and PI358610 (P. sativum abyssinicum).

g Assembled using CDC Bronco reads only.

h High- and low-copy contigs were identified based on comparison of the Unigene set with pea genomic sequences.

i JI2202 is a P. sativum abyssinicum accession; JI1703, a P. sativum elatius accession; and DCG076, a P. fulvum accession.

j Only reads from Cameor (n = 1,061,737,954) were used to generate a gene-space assembly of the pea genome.

N Normalization of cDNA libraries.

Uncovering the molecular bases underlying agriculturally important traits requires knowledge of the gene content of genomic regions controlling these traits of interest. Besides recently available genomic resources such as the pea gene atlas (Alves-Carvalho et al., 2015), whole-genome polymorphism data for multiple genotypes (see Table 2 for full description), BAC libraries developed for the genotypes Cameor (http://cnrgv.toulouse.inra.fr/fr) and PI 269818 (Coyne et al., 2007), researchers can count on the conserved synteny between pea and close species with available genome sequences. Gene-based rich individual and consensus maps have revealed connections between pea and *Medicago truncatula* (Choi et al., 2004; Aubert et al., 2006; Bordat et al., 2011; Leonforte et al., 2013b; Duarte et al., 2014; Sindhu et al., 2014; Tayeh et al., 2015a), *Lotus japonicus*, soybean (Bordat et al., 2011; Leonforte et al., 2013b; Tayeh et al., 2015a), pigeon pea (Leonforte et al., 2013b), chickpea (Leonforte et al., 2013b; Tayeh et al., 2015a), and lentil (Sindhu et al., 2014). Comprehensive understanding of shared syntenic blocks was, for instance, reported to be of great help to identify candidate genes for the control of seed size (D'Erfurth et al., 2012) and freezing tolerance (Tayeh et al., 2013a,b).

As other legume crops, pea is not easily amenable to genetic transformation (Warkentin et al., 2015): transformation occurs at low rates (Svabova and Griga, 2008) and plant regeneration is difficult. Fortunately, functional validation of candidate genes can benefit from a TILLING population developed from the genotype Cameor (Dalmais et al., 2008) and from the Virus Induced Gene Silencing (VIGS) methodology successfully adapted in pea (Grønlund et al., 2010; Pflieger et al., 2013).

## The use of genomic tools in marker-trait association studies and breeding programs

Genetic maps have proven to be useful to uncover the molecular bases of monogenic characters such as Mendel's characters (see Ellis et al., 2011 for review) and also to decipher the determinism of complex agronomically-important traits (Table 1). QTLs responsible for the genetic control of yield-related traits, seed protein content, aerial and root architecture, and biotic and abiotic stress resistance have been detected under multiple environmental conditions and located on different maps (Table 1). In addition to QTL mapping analyses in bi-parental populations, association analyses have emerged as a complementary approach to dissect quantitative traits in pea by exploiting natural genetic diversity and ancestral recombination events characterizing germplasm collections. Diverse sets of cultivars with distinct geographic origins were used to determine associations between genetic markers and seed mineral nutrient concentration (Kwon et al., 2012; Cheng et al., 2015; Diapari et al., 2015), seed low-carbohydrate content (Cheng et al., 2015), seed lipid content (Ahmad et al., 2015), yield-related traits (Kwon et al., 2012), disease/pest resistance, and morphological traits such as flower color and seed coat color. Genome wide association mapping and subsequent allele mining could build on genomic resources reviewed herein and the phenotyping data available for diverse germplasm collections, as reviewed in Warkentin et al. (2015).

Specific markers linked to major genes were developed for use in breeding, especially for trypsin inhibitors in pea seeds (Page et al., 2002; Duc et al., 2004), flowering (Weller and Ortega, 2015), lodging resistance (Zhang et al., 2006) and resistance to diseases such as powdery mildew (Ghafoor and McPhee, 2012; Reddy et al., 2015), pea enation and seed borne mosaic virus (Frew et al., 2002; Jain et al., 2013), fusarium wilt (McClendon et al., 2002), Ascochyta blight (Jha et al., 2015), and rust (Barilli et al., 2010) (Supplementary Table 1). Some other resistance, flowering or seed composition genes were reviewed by Warkentin et al. (2015). Marker-assisted selection was conducted in early generation (F_2_) breeding populations using markers linked in coupling to two major QTLs controlling lodging resistance and was demonstrated more efficient than phenotypic selection (Zhang et al., 2006). Recently, marker-assisted backcrossing (MABC) was successfully used to introgress one to three of the seven main *Aphanomyces* root rot resistance QTLs (Hamon et al., 2013) into several recipient agronomic lines (Lavaud et al., 2015). Evaluation for resistance of the subsequent 157 BC5/6 Near Isogenic Lines (NILs) validated the effect of the major and some minor QTLs in controlled conditions and showed QTL x genetic background interactions. A MABC strategy was also used to introgress three frost tolerance QTLs among the main four QTLs identified by Lejeune-Hénaut et al. (2008). Field evaluations of 125 QTL-NILs validated the effect of these QTLs in the spring-type genetic background Eden (Hascoët et al., 2014). So far, marker-assisted construction of QTL-NILs has mainly allowed QTL effects to be validated. The rational use of these genetic regions in breeding strategies can now be considered in order to combine favorable alleles at complementary QTLs to improve multiple stress resistance in agronomic material. A list of the markers that should be useful for pea breeding is provided in Supplementary Table 1. In parallel to strategies considering the combination of individual QTLs, genomic selection seems a promising approach in pea, as first suggested by the prediction of the date of beginning of flowering and 1000 seed weight using a subset of 331 SNP markers genotyped in a reference collection of 372 pea accessions (Burstin et al., 2015). Increasing the marker coverage of the genome by using the newly-developed GenoPea 13.2K SNP Array (Tayeh et al., 2015a) further improved prediction accuracies (Tayeh et al., 2015b).

## Perspectives

The gradually-developed genomic tools for pea now represent a rich resource for innovative strategies in both basic research and applied breeding. The large set of bi-parental interconnected populations segregating for diverse important agronomic traits, the individual and consensus genetic maps, the dense arrays of genetic markers, the high-throughput SNP genotyping tools, the BAC libraries, the TILLING population, and the whole-genome and transcriptome sequences from a large group of accessions should enhance significant advances in pea breeding in the next few years and foster the use of more diverse genetic resources for pea improvement. The genome sequence when released will further advance the pea genomic breeding revolution.

### Conflict of interest statement

The authors declare that the research was conducted in the absence of any commercial or financial relationships that could be construed as a potential conflict of interest.

## Footnotes

^1^FAOSTAT. http://faostat3.fao.org/. 10/06/2015.
