# Supplementary material for: Genomic Tools in Pea Breeding Programs: Status and Perspectives
Source: Front Plant Sci. 2015 Nov 27;6:1037. doi: 10.3389/fpls.2015.01037 (PMC4661580; doi:10.3389/fpls.2015.01037)
Supplement: Supplementary file 1 [file Table1.DOCX]

**Supplementary Table 1.** Markers for marker-assisted selection.

| **Trait** | **Locus Name** | **Markers (Name, Type, Gene-targeted or linked marker)** | **Reference(s)** |
| --- | --- | --- | --- |
| Seed trypsin inhibitor activity | *Tri* | Tri, composite PCR, gene-targeted marker | Page et al., 2002; Duc et al., 2004 |
| Resistance to powdery mildew | *Er* | Sc-OPO-18_1200_, RAPD-SCAR, linked marker | Ghafoor and McPhee, 2012 |
|  |  | OPL-6, RAPD, linked marker |  |
|  |  | OPD-10_650_, RAPD-SCAR, linked marker |  |
|  |  | Sc-OPD-10_650_, SCAR, linked marker |  |
|  |  | Sc-OPE-16_1600_, RAPD-SCAR, linked marker |  |
|  |  | P236, RFLP, linked marker |  |
|  |  | PSMPSAD60, SSR, linked marker |  |
|  |  | PSMPSAA374e, SSR, linked marker |  |
|  |  | PSMPA5, SSR, linked marker |  |
|  |  | ScX17_1400_, SCAR, linked marker |  |
|  |  | SCAB1_874_, SCAR, linked marker |  |
|  |  | BC210, RAPD-SCAR, linked marker |  |
|  |  | OPB18_430_, RAPD, linked marker |  |
|  |  | A5, SSR, linked marker | Reddy et al., 2015 |
| Resistance to pea enation virus | *En* | CNGC/tRNAMet, STS, linked markers | Jain et al., 2013 |
| Resistance to pea seed borne mosaic virus | *sbm1* | sG05–2537/sL01–910/sP446, RAPD/RFLP-derived STS, linked markers | Frew et al., 2002 |
| Resistance to fusarium wilt race 1 | *Fw* | ACG:CAT_222/ACC:CTG_159/Y15_1050, AFLP/RAPD, linked markers | McClendon et al., 2002 |
| Resistance to Ascochyta blight | *PsDof1* | PsDof1p308, SNP-KASP, gene-targeted marker | Jha et al., 2015 |
|  | *RGA* | RGA-G3Ap103, SNP-KASP, gene-targeted marker |  |
| Rust resistance | *Up1* | OPY11_1316_/OPV17_1078_, RAPD, linked markers | Barilli et al., 2010 |
| Lodging resistance | Lodging QTL on LG III | A001, AFLP-derived SCAR, linked marker | Zhang et al., 2006 |
|  | Lodging QTL on LG VI | A004, AFLP-derived SCAR, linked marker |  |
| Aphanomyces root rot resistance | *AePs1.2* | PD21-226/AC75-297, SSR, linked markers | Hamon et al., 2013; Lavaud et al., 2015 |
|  | *AePs2.2* | AA372.1-266/PD23-210, SSR, linked markers |  |
|  | *AePs3.1* | AA175-282/AD57-300, SSR, linked markers |  |
|  | *AePs4.1* | AA174-442/AD186.2, SSR, linked markers |  |
|  | *AePs4.2* | AA122-212/AC32-274, SSR, linked markers |  |
|  | *AePs5.1* | AA81-245/AB47-322, SSR, linked markers |  |
|  | *AePs7.6* | AA505-168/AB101-365, SSR, linked markers |  |
| Frost tolerance | *WFD3.1* | AA175, SSR, linked marker | Lejeune-Hénaut et al., 2008 |
|  | *WFD 5.1* | AD79, SSR, linked marker |  |
|  | *WFD 6.1* | AD59, SSR, linked marker |  |

Number of specific markers have also been developed for flowering genes (review in Weller and Ortega 2015) but their use in marker-assisted selection is not reported yet.

Abbreviations: LG, linkage group; QTL, quantitative trait locus; AFLP, amplified fragment length polymorphism; KASP, kompetitive allele-specific PCR ; RFLP, random fragment length polymorphism; RAPD, random amplified polymorphic DNA; SSR, simple sequence repeat; SCAR, sequence-characterized amplified region; SNP, single nucleotide polymorphism; STS, sequence-tagged site.
